# Spatial behavior of hepatitis A, MMR, and varicella vaccination coverage in the state of Minas Gerais, 2020

**DOI:** 10.1590/1980-549720230030

**Published:** 2023-06-23

**Authors:** Gabriela Cunha Corrêa Freitas de Oliveira, Luiz Henrique Arroyo, Aline Mendes Vimieiro, Josianne Dias Gusmão, Valéria Conceição de Oliveira, Eliete Albano de Azevedo Guimarães

**Affiliations:** I Universidade Federal de São João del-Rei – Divinópolis (MG), Brazil.; II Universidade de São Paulo – Ribeirão Preto (SP), Brazil.; III Minas Gerais State Health Department – Belo Horizonte (MG), Brazil.

**Keywords:** Immunization programs, Vaccines, Vaccination coverage, Health information systems, Spatial analysis, Public health nursing, Programas de imunização, Vacinas, Cobertura vacinal, Sistemas de informação em saúde, Análise espacial, Enfermagem em saúde pública

## Abstract

**Objective:**

To analyze the spatial behavior of hepatitis A, measles, mumps, and rubella (MMR), and varicella vaccination coverage in children and its relationship with socioeconomic determinants in the state of Minas Gerais.

**Methods:**

This ecological study investigated records of doses administered to children, extracted from the Immunization Information System of 853 municipalities in Minas Gerais, in 2020. We analyzed the vaccination coverage and socioeconomic factors. Spatial scan statistics were used to identify spatial clusters and measure the relative risk based on the vaccination coverage indicator and the Bivariate Moran Index, and thus detect socioeconomic factors correlated with the spatial distribution of vaccination. We used the cartographic base of the state and its municipalities and the ArcGIS and SPSS software programs.

**Results:**

Hepatitis A (89.0%), MMR (75.7%), and varicella (89.0%) showed low vaccination coverage. All vaccines analyzed had significant clusters. The clusters most likely to vaccinate their population were mainly located in the Central, Midwest, South Central, and Northwest regions, while the least likely were in the North, Northeast, and Triângulo do Sul regions. The municipal human development index, urbanization rate, and gross domestic product were spatially dependent on vaccination coverage.

**Conclusions:**

The spatial behavior of hepatitis A, MMR, and varicella vaccination coverage is heterogeneous and associated with socioeconomic factors. We emphasize that vaccination records require attention and should be continuously monitored to improve the quality of information used in services and research.

## RESUMO

**Objetivo:**

Analisar o comportamento espacial da cobertura vacinal da hepatite A, tríplice viral e varicela em crianças e a relação com determinantes socioeconômicos, no estado de Minas Gerais.

**Métodos:**

Estudo ecológico que considerou os registros de doses aplicadas em crianças extraídos do Sistema de Informação de Imunização de 853 municípios mineiros, em 2020. Analisaram-se as coberturas vacinais e os fatores socioeconômicos. A estatística *scan* espacial foi utilizada para identificar agrupamentos espaciais e medir o risco relativo pelo indicador de cobertura vacinal e o Índice de Moran Bivariado para identificar os fatores socioeconômicos correlacionados com a distribuição espacial da vacinação. Utilizou-se a base cartográfica do estado e de seus municípios e os *softwares* ArcGIS e SPSS.

**Resultados:**

Observaram-se baixas coberturas para as vacinas de hepatite A (89,0%), tríplice viral (75,7%) e varicela (89,0%). Foram identificados aglomerados significativos em todas as vacinas analisadas. Os aglomerados de maior chance de a população ser vacinada se alocaram majoritariamente nas regiões Centro, Centro-Oeste, Centro-Sul, Noroeste, e as de menor propensão para a vacinação encontram-se nas regiões Norte, Nordeste e Triângulo do Sul. O índice de desenvolvimento humano municipal, a taxa de urbanização e o produto interno bruto tiveram dependência espacial com a cobertura vacinal.

**Conclusões:**

Há heterogeneidades no comportamento espacial das coberturas para as vacinas hepatite A, tríplice viral e varicela, e essa condição se encontra associada a fatores socioeconômicos. Destaca-se que os registros de vacinação requerem atenção e devem ser continuamente acompanhados para aprimoramento da qualidade das informações utilizadas nos serviços e nas pesquisas.

## INTRODUCTION

Considered a priority intervention, vaccination prevents infant mortality and reduces hospitalization and vaccine-preventable diseases, avoiding up to 2.5 million deaths worldwide every year^1–3^. This intervention is regarded as one of the greatest achievements of humanity, recognized as the best cost-benefit public health investment^3,4^.

Currently, the Immunization Agenda 2030 global strategy foresees a world where people of all ages and places benefit fully from the vaccines offered to improve the population's health and well-being. This intervention proposes to maintain the positive vaccination results achieved and recover the losses caused by SARS-CoV-2 (COVID-19)^5^.

However, less than two-thirds of all countries have reached optimal coverage^6^. Among the vaccines offered to children, the measles, mumps, and rubella (MMR) and varicella vaccines are experiencing a decrease in coverage. These diseases are highly contagious and have several associated clinical complications^7^. The hepatitis A vaccine also stands out, with a drop in vaccination coverage in all Brazilian states after 2015^8^. The disease has mild clinical manifestations in childhood and rarely progresses into the severe condition of fulminant failure.

In 2020, about 23 million children missed essential vaccines — 3.7 million more than in 2019^5^. Around 3 million children did not receive the first dose of the measles vaccine, contributing to the increase in outbreaks, such as those that occurred in Venezuela (2017), Madagascar, the Philippines, and Brazil (2018 and 2019)^9,10^. Vaccination coverage has been dropping in European countries since 2016, with measles and diphtheria, tetanus, and pertussis (DTP) vaccines showing the highest decreasing rates, reaching almost 14 million children not fully immunized in 2019^11^. In Montana, United States, less than two out of five children aged 24 months are fully immunized with vaccines administered in childhood^12^.

In Brazil, vaccination coverage is not evenly distributed, with reduced rates for some vaccines offered in the National Immunization Program (*Programa Nacional de Imunizações* — PNI) schedule^13–15^. From 2006 to 2016, some states, such as Goiás, Mato Grosso, and Minas Gerais, had a reduced number of children vaccinated with MMR^15,16^. Since 2015, hepatitis A vaccination coverage decreased among Brazilian municipalities, ranging from 60% to 82%^8^. Varicella had a mean coverage of 78.0% in 2016, a rate that has been declining ever since, reaching 34.3% in 2019^17^.

Low vaccination coverage is often related to the population's geographic conditions and socioeconomic status^18–20^, structural conditions, supply and access to health services^14,19,21^, lack of awareness of the strategies recommended by the immunization program^22–24^, vaccine hesitancy^13,14^ and, more recently, the COVID-19 pandemic^25,26^. The last one has aggravated pre-existing health inequities, exposing social inequalities, discrimination, and health gradients in human populations, both between and within countries^27^.

In view of the COVID-19 pandemic, which further exacerbated the population's vaccination status, surveillance becomes particularly important and necessary to reduce non-vaccinated population clusters and, consequently, avoid the risk of new epidemics of vaccine-preventable diseases. Systematic monitoring of vaccination coverage is a crucial management action to know not only what motivates vaccine delays and refusals but also the realities involved in this process^19,28–30^.

Knowing the spatial coverage distribution and its possible determinants allows the identification of interfering factors, essential for planning and implementing effective vaccination strategies. The specific contribution of using spatial analysis in the vaccine field has been discussed for years and has steadily increased since the mid-2000s^31^. This analysis has been used in Brazil; however, it needs to be strengthened, given the country's continental dimensions, population size, cultural and socioeconomic diversity, and the foundations of its regional differences^32^.

Thus, this study aimed to analyze the spatial behavior of hepatitis A, MMR, and varicella vaccination coverage in children and its relationship with socioeconomic determinants in the state of Minas Gerais.

## METHODS

This ecological study sought to determine the hepatitis A, MMR, and varicella vaccination coverage and its spatial distribution in Minas Gerais, in 2020.

**Figure 1:**
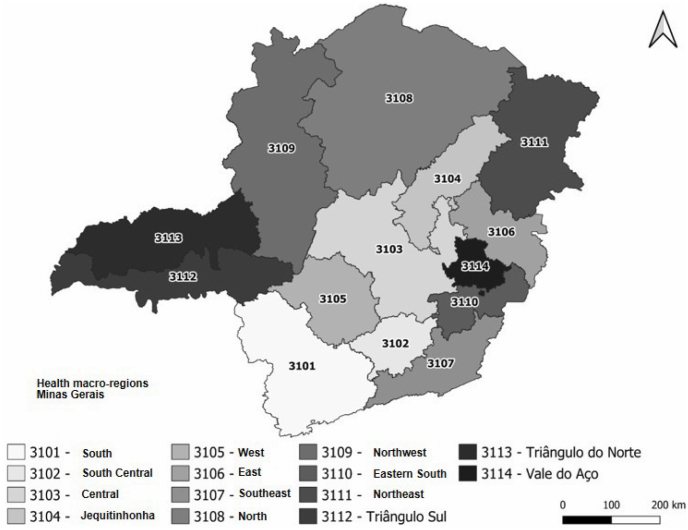
Minas Gerais macro-regions, Brazil, 2020.

Minas Gerais, Brazil's second most populous state, has an area of 586,528.293 km^2^, an estimated population of 20,033,665 inhabitants, and a degree of urbanization of 85.29%^33^. Its territory is divided into 14 macro-regions (South, South Central, Central, Jequitinhonha, West, East, Vale do Aço, Southeast, North, Northwest, Eastern South, Northeast, Triângulo do Sul, Triângulo do Norte), covering 853 municipalities^34^. This study established the municipalities of the 14 Minas Gerais macro-regions as territorial units of analysis (Figure 1).

We analyzed records — obtained from the PNI Information System database — of hepatitis A (single dose), MMR (second dose), and varicella (first dose)^35^ vaccine doses administered to 15-month-old children from the 853 municipalities of Minas Gerais, in 2020. A spatial analysis was carried out to identify and evaluate clusters of vaccinated children^36^ and possible socioeconomic factors associated with vaccination coverage. The MMR plus varicella (MMRV) vaccine was not included due to its shortage during the study period^37^.

Considered the response variable, vaccination coverage was calculated as the total doses for full immunization of each vaccine analyzed in the numerator and the number of live births in the municipality (recorded in the Live Birth Information System, in 2019) in the denominator, multiplied by 100. According to the Live Birth Information System, 256,892 children were born in the state in 2019^38^, the fraction corresponding to the denominator used to calculate the vaccination coverage indicator for 2020. The PNI set a 95% target for the hepatitis A, MMR, and varicella vaccines^37^.

The explanatory variables analyzed were grouped based on socioeconomic variables (municipal human development index; urbanization rate; gross domestic product *per capita*), extracted from the Brazilian Institute of Geography and Statistics and the João Pinheiro Foundation^39^ (Chart 1).

**Chart 1:** Description of the variables analyzed in the study.

Variables		
Response variable		
	Vaccination coverage	Vaccination coverage was calculated as the total doses for full immunization of each vaccine in the numerator and the number of live births in the municipality (recorded in the Live Birth Information System) in the denominator, multiplied by 100. The National Immunization Program set the target of: 95% for MMR, hepatitis A, and varicella^37^.
Explanatory variables — socioeconomic variables		
	Municipal human development index	The municipal human development index is a measure comprising indices related to three dimensions of human development: life expectancy, education, and income. The index ranges from 0 to 1. The closer to 1, the greater the human development.^33^
	Urbanization rate	Urban population divided by the total population, multiplied by 100. The urbanization rate corresponds to the percentage of the urban population in each state compared to the total population^33^.
	Gross domestic product *per capita*	Total gross domestic product of the municipality in the year divided by its total population, in up-to-date reais^39^.

MMR: measles, mumps, and rubella.

Clusters of vaccinated children were spatially identified and analyzed using the spatial analysis technique called scan statistics, developed by Kulldorff and Nagarwalla^36^. This analysis involves a gradual scan based on analysis windows with varying sizes throughout the territorial extension of the studied scenario. To this end, this research defined a circular analysis window, with its radius having a specific upper limit, established at 50% of the target population of vaccinated children in Minas Gerais. The intrinsic characteristic of this window is its high flexibility in both location and size, producing an infinite number of distinct geographic circles, each of them eligible as a cluster^36^.

Scan statistics uses different probability models. For the number of vaccinated children in the municipalities of Minas Gerais, we adopted the discrete Poisson model, considered the most suitable for rate modeling or count data. Each window has its null hypothesis (H0) tested against the alternative hypothesis (H1) — high risk for the event investigated (vaccination coverage) — compared to the outside window, that is, the remaining territory analyzed^40^.

The analysis was processed by the SaTScan 9.6 software, setting non-overlapping clusters as parameter^41^. The significance test for the identified clusters was based on the comparison between likelihood ratio test statistics and a null distribution, according to the Monte Carlo simulation. A higher number of replications in the Monte Carlo simulation affects the power of the respective test; thus, 999 replications were established to analyze the vaccination in the state^36^.

We considered the relative risk (RR) for significant clusters as a way of comparing information from dissimilar areas. RR is a non-negative epidemiological measure that represents how common the event is in the given spatial cluster. Taking into account the number of vaccinated children in Minas Gerais, RR values greater than 1 (RR>1) correspond to an increased probability of being vaccinated at a certain location, while values less than 1 (RR<1) mean the opposite, that is, a lower likelihood of being vaccinated in a region^42^. This calculation is performed with the following mathematic formula:


$$RR=\frac{cE[c]}{\left(C-c)(E[C]-E[c]\right)}=\frac{cE[c]}{\left(C-c)/(C-E[c]\right)}$$

In which *c* is the number of cases inside the cluster, *C* is the total number of cases in the dataset, *E[c]* is the number of cases expected inside the window under the null hypothesis. We also calculated 95% confidence intervals (95%CI) of RRs of significant spatial clusters^43,44^.

Bivariate Moran analysis was conducted to identify socioeconomic factors correlated with the spatial distribution of vaccination in Minas Gerais. This analysis is a global autocorrelation measure that estimates the influence of one variable on a neighboring one or the correlation between them^45^.

The Bivariate Moran can be interpreted as a regression coefficient in a bivariate regression, in which p-values are generated with significant values (p<0.05)^45^. The variables selected to analyze spatial correlation were those presented in the method: municipal human development index; urbanization rate, and gross domestic product *per capita*.

Both the analysis of global spatial autocorrelation (Moran index) and the Bivariate Moran (spatial correlation) were based on a queen contiguity matrix, in which municipalities that share borders are considered neighbors. These analyses were conducted in the Geoda 1.12 software.

Choropleth maps with results from the respective scanning analyses were prepared using the cartographic base of Minas Gerais and its respective municipalities, freely obtained from the website of the Brazilian Institute of Geography and Statistics and elaborated in the ArcGIS 10.8 software.

The study used public domain data with unrestricted access and anonymity for the individuals participating in the investigation; therefore, it did not require appreciation by the Research Ethics Committee.

## RESULTS

**Figure 2:**
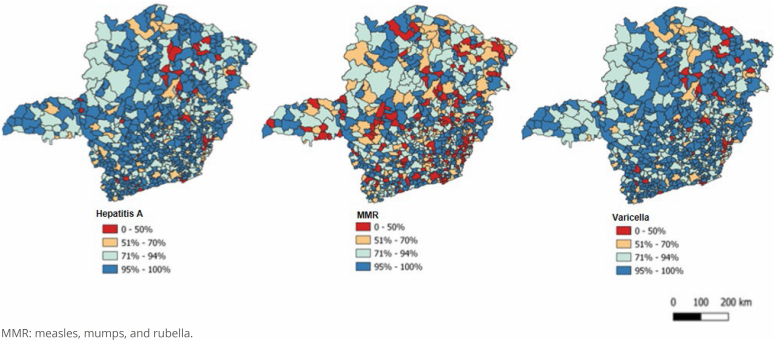
Spatial distribution of hepatitis A, MMR, and varicella vaccination coverage in Minas Gerais, 2020.

In Minas Gerais, the vaccination coverage for hepatitis A (89.0%), varicella (89.0%), and MMR (75.7%) was low in 2020. Coverage reached 95.0% or more in only 57.2% (n=488) of the municipalities for hepatitis A, 56.2% (n=479) for varicella, and 35.3% (n=301) for MMR. Figure 2 reveals similarities in the spatial distribution of hepatitis A and varicella vaccination coverage. This pattern was not identified in the distribution of the MMR vaccine, which had a higher number of municipalities with coverage below 95% (n=552).

**Figure 3:**
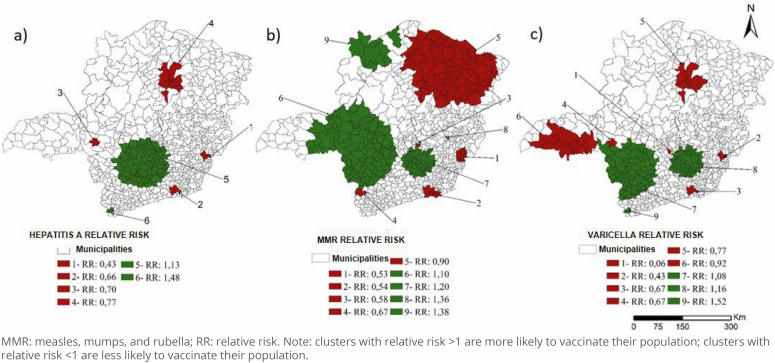
Areas with spatial clusters for hepatitis A, MMR, and varicella vaccination coverage in children under two years of age, Minas Gerais, 2020.

Spatial scan statistics detected statistically significant clusters for hepatitis A, MMR, and varicella vaccination coverage (Figure 3).

Clusters in the North and Northeast regions had the highest territorial extension (greater number of municipalities in the same cluster) and a lower likelihood of vaccinating their population with hepatitis A (cluster 4), MMR (cluster 5), and varicella (cluster 5) vaccines. The Triângulo do Sul region also presented a cluster with lower odds of vaccinating children against varicella (cluster 6). In contrast, the Central, Midwest, South Central (hepatitis A: cluster 5; MMR: cluster 6 and 7; varicella: cluster 7 and 8), and Northwest regions (MMR: cluster 9) showed the opposite behavior, that is, higher likelihood of vaccinating the population.

The hepatitis A vaccine had six clusters, four of them less likely to vaccinate the population. The MMR and varicella vaccines produced nine clusters. Both showed more clusters with lower odds of having their population vaccinated. Clusters with both higher and lower vaccination likelihood presented a similar pattern; namely, they were in neighboring regions, despite having different sizes, allowing the assumption that some characteristics lead municipalities to have similar coverage.

Table 1 shows results from analyses of the vaccination coverage in 2020 and the socioeconomic variables selected for the study in an attempt to identify factors that may have influenced the change in coverage values for the hepatitis A, MMR, and varicella vaccines in exploratory terms. The municipal human development index variable had a highly statistically significant positive spatial correlation with the coverage of the three vaccines analyzed. This result indicates that municipalities with higher percentages of vaccination coverage tend to be spatially correlated when municipal human development indices are higher in neighboring municipalities.

**Table 1:** Socioeconomic factors associated with hepatitis A, MMR, and varicella vaccination coverage in Minas Gerais, 2020.

Socioeconomic variables	Global Bivariate Moran Index		
	Hepatitis A I (p-value)*	MMR I (p-value)*	Varicella I (p-value)*
Municipal human development index	0.089 (0.001)	0.032 (0.017)	0.091 (0.001)
Urbanization rate	0.057 (0.002)	0.021 (0.100)	0.057 (0.003)
Gross domestic product *per capita*	0.033 (0.009)	0.005 (0.410)	0.032 (0.012)

* Significant values (p<0.05). MMR: measles, mumps, and rubella.

Also, the urbanization rate and gross domestic product *per capita* variables showed a low positive spatial correlation with hepatitis A and varicella vaccination coverage. We underline that, despite having a similar but very low trend in positive spatial correlation with these indices, the MMR vaccination coverage displays an important uniqueness in its spatial distribution. This finding is a strong indication that some external factor is influencing this spatial distribution. Nonetheless, this external factor was not included in this analysis, resulting in a different Bivariate Moran value for the hepatitis A and varicella vaccines.

## DISCUSSION

The spatial analysis of vaccination coverage in pandemic times pointed to a heterogeneous behavior of the hepatitis A, MMR, and varicella vaccines among the macro-regions in this Brazilian state, a condition associated with socioeconomic factors.

Regional coverage variations are also found in Brazil and other countries. Such differences result from significant geographic, social, and cultural discrepancies between and within regions^16,46–50^. Brazil and Minas Gerais, for example, have large territories with unequal socioeconomic effects among their population groups^51^. This inequality directly affects access to and care in health services, especially for the poorest or most vulnerable populations^52^. The need for better governance regarding interdependencies between health, social, environmental, and economic systems to ensure public health equity has been increasingly recognized worldwide^27^.

This study reveals a greater spatial correlation between vaccination coverage and the municipal human development index, a measure comprising indices related to three dimensions of human development: life expectancy, education, and income.

In Spain, children whose parents had a higher level of education were more likely to be vaccinated^53^. In Nigeria, a higher household income contributed to the children's full immunization^54^. In Brazil, studies report that the higher the level of education^55,56^ and income of parents^8,57,58^, the lower the probability of full immunization. This scenario may be associated with vaccine hesitancy^8,26,57,59^, which was more evident during the COVID-19 pandemic and exposed concerns about the quality and safety of vaccines^8,26,59^.

Gross domestic product and urbanization rate affect vaccination coverage and other socioeconomic factors because they can contribute to a poverty scenario^8,50^, thus impacting the organizational and geographic access to health services and their quality^16,18,19,22,60,61^. Brazilian studies have identified that the place of residence influences the drop in vaccination coverage^57,62^.

However, in addition to the socioeconomic inequalities detected in this study, other determinants associated with low vaccination coverage have been evidenced, such as difficult access to health services^22,63^, number of children^64,65^, missed vaccination opportunities^66,67^, underfunding of the health sector, and the complexity of the immunization schedule^14^. The COVID-19 pandemic has also aggravated pre-existing social inequalities in health, exposing deeply rooted social shortcomings, discrimination, and health gradients in human populations, between and within countries^27^.

We can assume that an external factor is influencing the MMR vaccination coverage distribution. This factor may be related to the record of doses administered in the Brazilian Immunization Information System. The system change in Brazil (2014) altered how the system is populated, now based on nominal entries, complicating the process of recording the doses administered^68,69^. Currently, the doses administered are recorded in the Citizen's Electronic Health Record, software developed by the e-SUS Primary Health Care strategy^35^.

The Brazilian child immunization schedule recommends an MMR dose at 12 months of age and a second MMR dose associated with the varicella component (MMRV) at 15 months^37^. However, the MMRV vaccine is not systematically distributed to all Brazilian states. Minas Gerais has not received a supply of this vaccine since 2018^70^. We can infer that some professionals might be recording a second MMR dose in the MMRV field. This assumption indicates underrated coverage, which agrees with the findings of this study, since the MMR vaccine showed a different spatial behavior from the other vaccines (hepatitis A and varicella), both administered at 15 months of age.

The use of the Immunization Information System became a complex and multidimensional process influenced by structural and process conditions, issues with technological components of the software, little experience of the health team with technological resources, and lack of training to use and understand the system, impacting the record of these data and, consequently, the vaccination coverage^68,71,72^.

Although this study aimed to provide an overview of vaccination coverage correlates, regional variability might occur within the municipalities and also among other cluster sets. Study limitations include its ecological design based on secondary data, which may have inconsistencies related to the quality and quantity of information, due to incorrect filling and mistaken records of doses administered in the information system. Nevertheless, the choice of this type of source reduces operational costs and enables the performance of analyses. A consistency analysis of the database was carried out to minimize this limitation.

The results of this article can help health managers and professionals design interventions to structure immunization services and implement management actions (supervision, monitoring, evaluation) to increase vaccination coverage in places at higher risk of transmission of vaccine-preventable diseases. The research field must advance the knowledge of the practice of professionals working in the vaccination station regarding surveillance actions related to the child's vaccination status and confirm, using other methodological designs, the factors that impact vaccination coverage in the child population.

In addition, vaccination records and the quality of data from immunization information systems are issues that require attention and must be continuously monitored to improve these data and reduce the entry of mistaken information.

Lastly, this study aims to provide the basis for policies and promote the equitable expansion of access to and use of immunization services. To that end, further research is necessary.
